# Progress with relationship continuity 2012, a British perspective

**DOI:** 10.5334/ijic.975

**Published:** 2012-06-29

**Authors:** George K Freeman

**Affiliations:** Emeritus Professor of General Practice, Imperial College London

**Keywords:** relationship continuity, continuity, primary care, patient experience

## Abstract

This perspective paper makes a brief conceptual review of continuity and argues that relationship continuity is the most controversial type. Plentiful evidence of association with better satisfaction and outcomes urgently needs to be supplemented by studies of causation. The scope of these has been outlined in this paper. Evidence strongly suggests that patients generally want more relationship continuity than they are getting and that relationship continuity is linked with better patient and staff satisfaction. This is reason enough to justify improving relationship continuity for patients.

## Introduction

Modern developments in Primary Health Care provision have led to increased interest in continuity of care. This is particularly notable in the UK, most likely because it is getting more difficult to provide continuity of care and hence for patients to enjoy it. When the level was high there was less interest, but now lower levels are leading to patients’ concern about the difficulty of seeing their preferred clinicians. Accumulating evidence that less continuity is associated with higher costs and poorer satisfaction and outcomes means that professionals and care commissioners should take this seriously.

### Changing emphasis over time

Uijen et al. have neatly outlined the history of interest in continuity in primary care over time [1]. In a review of the concept of continuity they point out how definitions and attitudes in general/family practice have evolved from the simple idea of a personal doctor in the 1950s. By the 1970s, various ways of measuring of how much patients saw the same doctor were accompanied by wider ranging exploration of the concept, with description of multiple dimensions such as chronological, geographical, interdisciplinary, interpersonal and informational continuity. By 1979 Starfield was referring to ‘continuous confusion’ [2]. These accounts of continuity took the professional viewpoint and while there were some notable trials in the US [3, 4], some of the best writing about continuity was theoretical and conceptual to guide the emerging discipline of general or family practice [5, 6].

My own interest in the topic arose from working in a group practice staffed by academic GPs who were all (necessarily) part-time clinicians, making it difficult for patients to consult the same doctor. We wondered if this really mattered, but an elegant student project—a simple compliance study—suggested that it did. Patients in our practice and in a neighbouring one were much more likely to complete a course of antibiotics if they felt they knew the prescribing doctor well. Importantly this association was stronger than that (also significant) with the number of times they had actually consulted that doctor [7]. I then worked in parallel with Per Hjortdahl in Oslo and by 1997 we were able to review a series of completed Norwegian and English studies and concluded that while there was evidence of continuity leading to better outcomes, the choice of clinician should be left to the patient, who should not be restricted to a single doctor [8], though this had seemed an attractive idea from the professional viewpoint [6].

### Importance of patient perspective

The new millennium has seen a welcome transfer of focus to the patient viewpoint. With increasing interest in patients’ experience of medical care, simultaneous continuity of care research programmes were funded in England and in Canada. Liaison between researchers scoping these programmes led to a joint conference and then a paper synthesising previous approaches to definition. The three types of continuity defined in this work by Haggerty et al. have informed much subsequent research [9]; its effect on the English programme has been elegantly described recently in this journal by Heaton et al. [10].

## Continuity of care

### Types of continuity

Continuity of care concerns individuals (rather than populations) over time. Three main types are *informational* and *management* continuity on the one hand, both of these referring to the co-ordination aspect of continuity, and *relationship* (relational) continuity on the other, concerning the patient’s on-going engagement with one or more clinicians [9]. Indeed some authors have formally merged these three types into two—information and management continuity making a ‘seamless service’ and a ‘continuous caring relationship’ being its interpersonal element [11]. Of these it is relationship continuity that is most difficult to achieve in modern medical care, while being more than ever necessary for the increasing number of patients with multi-morbidity [12, 13].

IJIC readers may feel that management and informational continuity (the ‘seamless service’) are closest to their interest, and some recent Canadian work has focused on this [14]. But other studies in that same programme remind us that these three continuity types are only constructs acting as aids to understanding. In the field there is considerable overlap between them [15]. For instance, patients can experience continuity as “connectedness between their personal lives and the health system” [16]; such perceptions go beyond coordination and link management with relationship continuity.

### Benefits of relationship continuity

In fact recent work on both sides of the Atlantic augments the evidence [17] that relationship continuity is indeed associated with better health outcomes. In Canada, Menec et al. showed how seeing the same primary care physician more than 75% of the time was associated with reduced odds of ‘ambulatory care sensitive’ hospitalization [18]. And in England, in a series of linked studies, Baker’s team has demonstrated associations of aspects of better continuity with lower rates of emergency hospital admission [19], and of elective hospital admissions [20]. Thus it is likely that the role of relationship continuity may currently be seriously underestimated in our search for affordable and effective health care.

### Decline of relationship continuity—can this be reversed?

As there is evidence of continuing decline in relationship continuity in England, particularly since the 2004 pay for performance contract [21], it is becoming urgent to try and reverse this trend. The case for this would be strengthened by clearer evidence for causation rather than just association. In other words, would improving continuity really improve outcomes? Answering this question requires intervention studies—of complex interventions studied by multiple methods—consuming both time and money. Given the long history of uncertain evidence, it is surely time to get started! But in the meantime, in order to even design an appropriate intervention, we need a better understanding of how continuity works—what actually happens in primary care?

## How does continuity work?

### How does continuity work?—continuity and access

It is well understood that relationship continuity is in an intimate trade-off with access [22]. Problems with primary care access caught the attention of British care planners at the turn of the millennium, and a series of initiatives led to incentivisation through access targets in the 2004 ‘pay for performance’ contract. These are still in place, but are not balanced by any matching emphasis on relationship continuity; this may account for its subsequent decline as already noted [21]. The 2004 changes also included registering patients with the practice as a whole, rather than with a specific doctor, and ending practices’ responsibility for 24 hour care; out of hours care is now the responsibility of Primary Care Trusts. Baker’s team has suggested another factor—the 2004 contract’s emphasis on chronic disease detection may have restricted access [23], making it more difficult for patients to see their chosen clinician. What is clear, both from qualitative studies [24, 25] and a series of discrete choice experiments [26–29], is that patients value access to a known and trusted practitioner when their problem is more serious or personal, and quicker access to any clinician when they perceive the problem as more acute or technical.

### How does continuity work?—patient experience

We still lack understanding of how patients get continuity (or not) when they want it. Some insight was given by Boulton et al., who found a wide spectrum of patients’ attitudes and wants, these in turn sometimes considerably modified by experience [30]. More work is needed to find out how patients achieve continuity in today’s context, which includes a range of buildings, appointment systems, staff attitudes and expertise, and varying clinician availability. Certain groups particularly merit study.

One such group is new patients, who necessarily cannot know and trust any clinicians in the practice (though of course they may join because of a personal recommendation). The English pay for performance scheme allows for a 50% increase in consultations for the first year after registration [31]. How do patients actually establish therapeutic relationships with one or more practitioners after joining a practice? Such a group could be recruited when they register and be followed-up by interview, telephone or diaries for 12–24 months.

A second group, perhaps more needy, comprises patients with a new diagnosis of chronic/serious illness. Again, these can be recruited at diagnosis and followed-up over time.

A third group is patients disadvantaged socially, educationally, or mentally, as highlighted by Baker et al. [32]. For example one West London GP is initiating work in his practice to find out how much continuity patients with a label of ‘personality disorder’ are getting (D Wingfield, personal communication).

A contrasting group could comprise patients already attached to a known and trusted clinician. What happens to them when their doctor leaves? (Anecdotes tell that some apparently rather ‘dependent’ patients cope surprisingly well!)

### How does continuity work?—how can practices improve?

Even if the need for improvement is agreed and priority groups of patients are identified, the interactions between the working of appointment systems, availability of clinician time, the scope of alternatives to face-to-face consultation such as telephone and email, and attitudes of practice staff are still little understood [33–35]. The key events happen between patients and practice receptionists either face-to-face (e.g. for a follow-up appointment) or on the telephone. This interface, arguably crucial for good care delivery, has attracted remarkably little attention. Current plans to bypass receptionists by direct on-line booking are interesting, but risk further disadvantaging the third group of patients (above). So action research on the implementation of continuity improvement programmes is needed. In the USA, Kibbe et al. reported a successful programme nearly 20 years ago [36] but there are important differences in Britain and other European countries where, among other things, capacity constraints are generally tighter and the gatekeeping role far more salient.

### How does relationship continuity improve outcomes?

**Figure 1:**
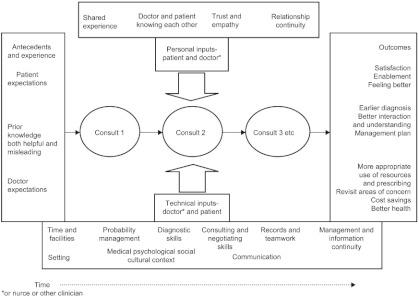
Personal and technical inputs to consultations and their links with types of continuity over time (after Freeman et al. 2003) [37].

If making it easier for patients to get relationship continuity significantly improves health outcomes, then we need to understand how this works. Freeman et al. proposed a simple model in 2003 (Figure 1).

The key feature is that patient inputs (including shared experience and trust) combine with professional expertise and resources, leading to outcomes ranging from satisfaction and enablement to earlier diagnosis, more appropriate management and better use of resources. These and other factors need to be monitored in complex intervention trials.

### The cancer diagnosis question

In relation to diagnosis, Vedsted and Olesen have recently implied the unwelcome possibility that relationship continuity, or at least an important primary care gatekeeping role linked with a patient list system, could actually delay diagnosis [38]. They suggest, for example, that early reassurance from a trusted clinician could falsely reassure a patient with a serious diagnosis and lead to delayed re-presentation if symptoms persist [39]. Certainly cancer detection rates in the UK have given rise to concern, appearing less good than in some other countries of comparable wealth. It is therefore highly appropriate to encourage research on the process of diagnosis in primary care with special reference to patients who may have cancer, and to the possible role, whether positive or negative, of relationship continuity.

## How relevant is Starfield’s work in the UK context?

Over the past 20 years, much advocacy of primary care in general and the role of the family doctor in particular has rested on the evidence of Barbara Starfield and her colleagues, best summarised in her classic book [40] and landmark Millbank Quarterly review of 2005 [41]. Starfield is very persuasive, but it must be said that the bulk of her primary evidence originates in the North American setting. This evidence is then interpreted in the context of comparative international routine data. While primary care continues to have such a limited role in the USA, this argument is highly relevant. But it may be stretching the point too far to use her evidence to make a case for improving relationship continuity in the UK. The studies suggested here could start to fill this gap.

## Conclusion

I have made a brief conceptual review of continuity and argued that relationship continuity is the most controversial type. Plentiful evidence of association with better satisfaction and outcomes urgently needs to be supplement by studies of causation. I have outlined the scope of these.

Meanwhile we should all remember that while many younger, fitter patients may neither need nor wish to see the same clinician each time, the situation is often quite different for older or more dependent patients, who may suffer multi-morbidities [13] and particularly benefit from a clinician that knows them well. Large-scale data from the National General Practice Patient Survey in England strongly suggest that patients generally want more relationship continuity than they are getting. This, combined with the overwhelming balance of evidence linking relationship continuity with better patient and staff satisfaction, is reason enough to justify improving relationship continuity for patients [42, 43].

## About the author

The author was an academic general practitioner in Southampton for 20 years before coming to London as the first professor of general practice at Charing Cross and Westminster Medical School in 1993. This School merged with Imperial College in 1997.

In Southampton it was a ‘most peculiar practice’ where all the GPs were full-time academics and therefore necessarily part time in the practice. The resulting continuity problems stimulated his MD thesis on Continuity of Care in General Practice. Work on Continuity has inevitably led to interest in wider aspects of quality of care including access, consultation length, patient enablement, and most recently generalism in medicine. Patients and professionals must often decide how to trade off seeing the right person against waiting for them.

With an international reputation in the field of continuity of care he collaborates with a wide range of colleagues, currently in Montreal, Leicester, and Manchester. Retiring from Imperial College in 2004, he was visiting Professor of General Practice at St George’s 2005–9. In 2010, together with Jane Hughes, he wrote the *Continuity of Care* report for the King’s Fund Inquiry *Improving the quality of care in general practice*. The following year he was asked to join the Royal College of General Practitioners’ *Commission on Generalism* and co-authored (with Alison Hill) the College’s Policy Paper *Promoting Continuity of Care in General Practice.*
